# Vagus nerve stimulation as an anti-inflammatory therapy for maternal immune activation-induced alterations in offspring microglia and neurodevelopment

**DOI:** 10.3389/fnins.2026.1827714

**Published:** 2026-04-24

**Authors:** Makenna Gargus, Linnea Poyhia, Joseph P. Errico, M. Karen Newell Rogers, Marie-Eve Tremblay

**Affiliations:** 1 Faculty of Health, School of Medical Sciences, University of Victoria, Victoria, BC, Canada; 2 Vagus Nerve Society, Atlantic Beach, FL, United States; 3 Department of Medical Physiology, Naresh K. Vashisht College of Medicine, Texas A&M University, Bryan, TX, United States; 4 Department of Biochemistry and Molecular Biology, The University of British Columbia, Vancouver, BC, Canada

**Keywords:** autism spectrum disorder, cytokines, interleukin-6, maternal immune activation, microglia, vagus nerve stimulation

## Abstract

Autism spectrum disorder (ASD) is a common neurodevelopmental disorder caused by a combination of genetic and environmental factors. With the increasing prevalence of ASD diagnosis, it is crucial to understand the mechanisms behind preventable causes, such as prenatal infections, and look for possible routes to improve outcomes. For example, maternal immune activation (MIA), the process by which immunogens that enter the maternal system lead to a maternal inflammatory response, has been well established as associated with increased ASD diagnosis. However, the mechanisms have not been fully elucidated and the options for targeting MIA as a preventative measure are uncertain. The maternal cytokine response is considered a crucial mechanism underlying MIA-induced neurodevelopmental changes, with key contributing cytokines, which include interleukin (IL)-6 and IL-17a. These cytokines can be produced in the maternal periphery and placenta, leading to the transmission of maternal cytokines into the fetal brain and causing upregulation of endogenous production. In the fetal brain, IL-6 and IL-17a act on microglia, the innate immune cells of the central nervous system, to further induce pro-inflammatory cytokine production. Furthermore, microglia alter fetal brain neurocircuitry, leading to lifelong, ASD-like dysregulation. The vagus nerve, the primary nerve of the parasympathetic nervous system, may serve as a target for intervention. The cholinergic anti-inflammatory pathway can be targeted by vagus nerve stimulation (VNS) and can lead to the downregulation of peripheral cytokines. This review is intended to summarize the cytokine-related mechanisms of MIA, the role of fetal microglia in dysregulation, and to assess the potential for VNS as a preventative treatment measure for MIA-induced alterations.

## 1 Introduction

Autism spectrum disorder (ASD) is a neurodevelopmental disorder that can lead to impairments in social communication and learning, as well as repetitive behaviors, limited interests, and increased anxiety and fear responses (Shivaswamy et al., 2022). In 2022, the Centers for Disease Control and Prevention reported the prevalence of ASD in the USA to be about 1 in 31 (3.2%) in children aged 8 years (Shaw, 2025). It is estimated that ASD is 40–80% genetic, while the remaining cases are believed to be due to environmental risk factors, such as increased parental age, exposure to pollution or toxins, diet, stress, and prenatal infections (Rylaarsdam and Guemez-Gamboa, 2019).

Prenatal infections, alongside risk factors such as psychological stress, autoimmune conditions, and exposure to environmental toxins (O’Connor and Ciesla, 2022), strongly influence the risk of maternal immune activation (MIA), an epidemiological term referring to maternal exposure to infection with immunogens (e.g., bacterial, viral), leading to a maternal immune response (Hall et al., 2023). Maternal infection alone is not responsible for ASD, as many instances of maternal infection do not lead to ASD, and is instead believed to be part of a multi-hit system, combining risk genes, epigenetic alterations, various maternal exposures, and early life environmental risk factors (Picci and Scherf, 2015). Additionally, the specific viral or bacterial infection involved does not seem directly linked either, as cases have emerged from a variety of infectious agents (Picci and Scherf, 2015). The mechanisms of MIA are not fully established, but many central theories focus on the role of the maternal inflammatory response, cytokine infiltration of the placenta, and the upregulation of fetal cytokine production, all of which could result from a variety of infections, exposure to toxins, maternal autoimmunity, or maternal stressors (Hall et al., 2023; Mahic et al., 2017; Shi et al., 2005). Additionally, in the fetal brain, cytokines can be upregulated and lead to self-propagating inflammation once they enter the brain through interactions with microglia (Guma et al., 2019; Ginhoux et al., 2010).

Microglia, the immune cells of the central nervous system (CNS), are derived from the primitive yolk sac and migrate to the fetal brain around embryonic day (E) 9.5 in mice (Guma et al., 2019; Ginhoux et al., 2010). Homeostatic balance in the CNS is primarily mediated by microglia (Tremblay, 2020). During the early stages of development, microglia play a role in numerous key processes, including promoting neurogenesis, neuronal circuit refinement, and the apoptosis of neural precursor cells to limit cell populations (Tremblay, 2020). When entering the developing cerebral cortex, microglia remain in the deeper layers for a time and assist with axon extension and laminar positioning (Squarzoni et al., 2014). Once cortical neurogenesis concludes, microglia are distributed evenly throughout the cortical layers (Cunningham et al., 2013). During the first two postnatal weeks in mice, microglia proliferate rapidly, then decline in number by more than half by postnatal week six (Nikodemova et al., 2015). Being long-lived cells, any microglial dysfunction during developmental critical periods can lead to lifelong consequences on brain circuitry and behavior (Tay et al., 2017; Prinz et al., 2021).

As resident immune cells, microglia are critical for CNS defense. Following acute insults, microglia rapidly extend their processes toward damage, phagocytose debris and pathogens, and release cytokines to propagate inflammatory responses (Sierra et al., 2014). Microglia also critically regulate many aspects of early brain development (Tay et al., 2017), including neuronal circuit maturation (Paolicelli et al., 2011), axonal outgrowth and laminar positioning (Squarzoni et al., 2014), and neuronal survival (Ueno et al., 2013). The functions of microglia continue and expand throughout development, including maintenance of synapses and plasticity, and regulation of neuronal activity (Paolicelli and Ferretti, 2017; Tremblay, 2021). Single-cell RNA sequencing studies have found that during early postnatal development in mice, microglial populations are more heterogeneous and less homeostatic compared to adulthood (Li et al., 2019; Hammond et al., 2019). In this period, microglia highly express factors associated with cellular metabolism, acting as a metabolic sensor, and as well as factors for proliferation and motility, which are often upregulated during injured conditions (Hammond et al., 2019).

Microglia are among the most downstream effectors for prenatal MIA, with dysregulation following exposure to the maternal disruptions. Cytokine levels are tightly controlled in the healthy CNS and are typically present at extremely low concentrations to serve homeostatic functions (West et al., 2019). For example, postnatal microglia inhabiting the subventricular zone promote neurogenesis and oligodendrogenesis by secreting a multitude of pro-inflammatory cytokines, namely interleukin (IL)-6, IL-1β, and TNF-α (Shigemoto-Mogami et al., 2014). Further, microglia can use cytokines such as IL-10 as part of the maintenance of synaptic development and plasticity (Andoh and Koyama, 2021). Local and transient increases in cytokine levels mediate effective immune responses to resolve CNS homeostasis after disruption, but chronic elevation can be damaging (West et al., 2019). This chronic elevation is exacerbated by MIA and contributes to the neurodevelopmental abnormalities seen after MIA (Mattei et al., 2017; Ozaki et al., 2020). The loss of this balance can alter microglial functions, as seen in ASD and schizophrenia by adulthood (Andoh and Koyama, 2021).

The vagus nerve (VN) is the tenth of our 12 paired cranial nerves, serving as a mixed sensory-motor nerve and as the primary parasympathetic component of the nervous system. Peripheral information is picked up from the VN’s afferent projections, which can be found abundantly innervating the stomach, as well as many other organs, such as the liver, kidneys, pancreas, and upper intestine (Gargus et al., 2025). Sensory stimuli are sent to the nucleus tractus solitarius, where they are then distributed across the hippocampus, thalamus, rostral ventrolateral medulla, amygdala, and cerebral cortex for further processing (Gargus et al., 2025). Motor signals are then sent back through the dorsal motor nucleus of the vagus (Gargus et al., 2025). These signaling processes modulate pro-inflammatory cytokine production through the cholinergic anti-inflammatory pathway (Gargus et al., 2025).

Vagus nerve stimulation (VNS) is a neuromodulation technology, most commonly surgically implanted to electrically stimulate the left cervical VN (Gargus et al., 2025). Alternative options include non-invasive forms, such as auricular VNS, which targets the branches of the VN at the cymba conchae, as well as cervical VNS, which applies electrodes to the surface of the neck to target the cervical VN (Gargus et al., 2025). Currently, VNS is an approved treatment for epilepsy and major depression disorder, while non-invasive forms are also used to treat chronic migraines (Gargus et al., 2025; Silberstein et al., 2016; Hawkins et al., 2017; Dawson et al., 2021; Fisher et al., 2020). VNS has been demonstrated to decrease pro-inflammatory cytokine production, at least in part via the cholinergic anti-inflammatory pathway (Kelly et al., 2022), and could provide a promising strategy for modulating the maternal inflammatory response in MIA (Liu et al., 2024).

In this review we will discuss (1) what is known about the mechanisms of MIA and summarize the important cytokines believed to be involved, (2) discuss microglia as a key cellular player in the mechanism of fetal brain alterations associated with MIA and a marker for MIA-induced effects, and (3) assess the potential for VNS as a treatment strategy for mothers to reduce peripheral maternal cytokine levels and decrease symptom severity in offspring exposed to MIA. Thus, our overall goal is to evaluate the currently understood mechanisms of MIA at the maternal level, how the fetal brain responds to these changes as a potential marker for ASD, and how we might apply VNS as a treatment strategy to prevent these alterations from occurring.

## 2 Maternal immune activation

The first evidence connecting MIA to neurodevelopmental dysregulation was published in 1988 by S. Mednick, who discovered that offspring exposed to influenza during the prenatal period developed schizophrenia at a higher rate (Mednick et al., 1988). More recently, a report from the UK Millennium Cohort found that maternal-reported infections were associated with increased odds of ASD, but did not find an association with ASD in hospital-reported infections (Hall et al., 2022). A meta-analysis by Jiang and colleagues in 2016 conversely found increased odds of ASD after exposure to any prenatal infection, and a stronger association when including only infections requiring hospitalization (Jiang et al., 2016). Studies have linked numerous viral infections during pregnancy with ASD diagnosis (e.g., rubella, influenza) in offspring, as well as bacterial infections (e.g., urinary tract infections, respiratory infections) (Hall et al., 2023; Massrali et al., 2022). Different infectious agents may contribute to differing associated risks, but studies have also noted an influence of timing, with some studies only reporting increased risk when infections occurred in the second trimester in humans (Hall et al., 2022). Outcomes of MIA have also been associated with other neurodevelopmental disorders, such as schizophrenia, major depressive disorder, and attention-deficit/hyperactivity disorder (Choudhury and Lennox, 2021; Khan et al., 2014; Schaer et al., 2026). The variance in neurodevelopmental disorder outcome can be associated with the timing of infection, as well as baseline susceptibilities and second hits (Stoltenberg et al., 2010). Further, many of these conditions have high comorbidity and may be present together (Bonti et al., 2024).

Several animal models of MIA have been developed to study its mechanisms and neurodevelopmental outcomes. Mice and rat models are common and can have slightly varying outcomes between species (Babri et al., 2014). Further, even within a species, such as mice, the strain of mouse used can lead to varying outcomes (Babri et al., 2014). Common models of infection-induced MIA utilize immunogens such as influenza, lipopolysaccharide (LPS; a bacterial endotoxin that mimics bacterial infection), and polyinosinic:polycytidylic acid (polyI:C; an mRNA viral mimetic), typically administered to pregnant rodents at timepoints corresponding to mid-pregnancy (E9.5 or E12 in mice) or late pregnancy (E17 in mice) (Meyer et al., 2006; Enayati et al., 2012). These periods are chosen for studies because they are considered critical periods of brain development that correspond to the human early second and late second to early third trimesters, respectively (McEwan et al., 2023). Both LPS and polyI:C are considered Toll-like receptor (TLR) agonists as they are known to bind to TLR4 and TLR3, respectively. As such, polyI:C and LPS elicit an acute immune response, inducing inflammatory cytokine and interferon (IFN) pathways to stimulate the innate inflammatory response, which peaks at 3–4 h post-injection and resolves within 24–48 h (Hall et al., 2023; Cunningham et al., 2007).

**Figure 1:**
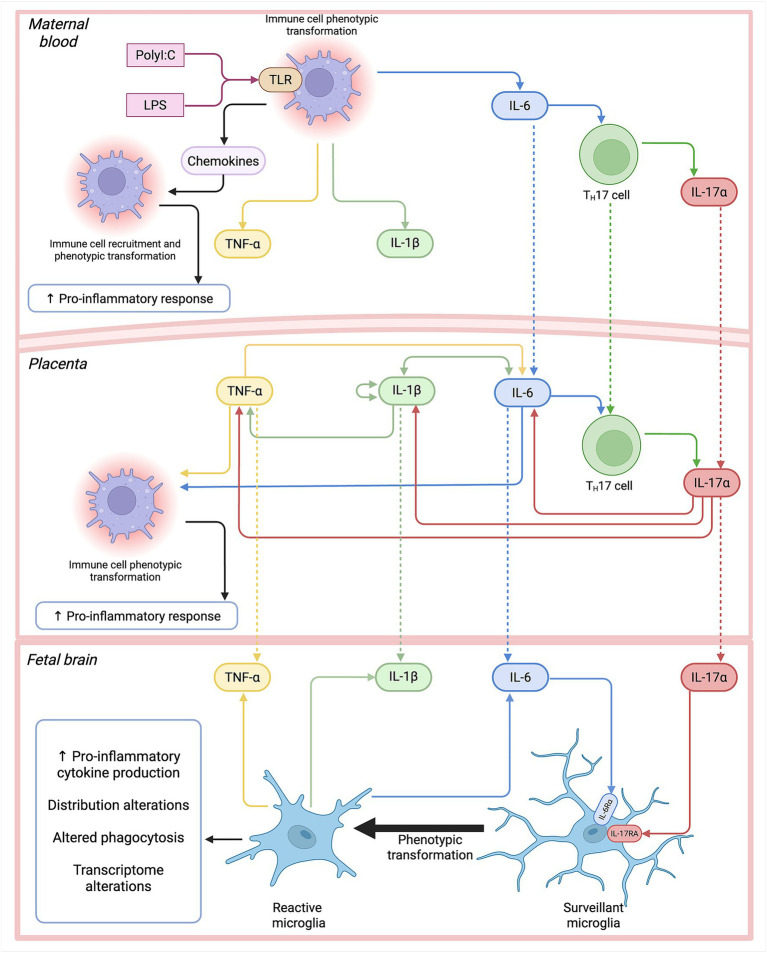
Cytokine pathways in maternal immune activation (MIA). In the maternal milieu, immunogens, such as lipopolysaccharide (LPS) and polyinosinic:polycytidylic acid (polyI:C), bind to Toll-like receptors (TLRs) on the surface of immune cells, leading to phenotypic transformation and the downstream stimulation of nuclear factor-κB (NF-κB), which increases production of pro-inflammatory cytokines, such as tumor necrosis factor alpha (TNF-α), interleukin (IL)-1β, and IL-6, as well as chemokines. IL-6 stimulates T helper cells 17 (T_H_17), which produce IL-17a. Only IL-6 and IL-17a can cross the placental barrier, where they influence the upregulation of pro-inflammatory cytokines and resident immune cell phenotypic transformation to upregulate the immune response. Pro-inflammatory cytokines can then all cross the blood–brain barrier and infiltrate the fetal brain. IL-17a and IL-6 can bind to IL-17 receptor A subunit (IL17RA) and IL-6 receptor alpha (IL-6Rα) on the surface of microglia, respectively, leading to stimulation of NF-κB, increased endogenous production of pro-inflammatory cytokines, increases in phagocytosis, and transcriptomic alterations. Schematic pathways are derived from listed references (Meyer et al., 2006; Liu et al., 2017; Zawadzka et al., 2021; Dahlgren et al., 2006; Zaretsky et al., 2004; Blank and Prinz, 2017; Choi et al., 2016; Tripathi et al., 2017; Fujitani et al., 2022; LaMonica Ostrem et al., 2024; Hui et al., 2018). Created with BioRender.com.

Dr. Paul Patterson’s lab originally proposed, and their thesis has since been supported by numerous studies, that the peripheral maternal cytokine-associated immune response increases the risk for ASD (Hall et al., 2023; Mahic et al., 2017; Shi et al., 2005). Cytokines comprise a number of proteins, such as IFNs, ILs, and transforming growth factors (TGFs), and broadly fall into two categories: pro-inflammatory and anti-inflammatory (Zhang and An, 2007). Some common pro-inflammatory cytokines that have been associated with MIA include IL-6, IL-17a, IL-1β, and tumor necrosis factor alpha (TNF-α) (Carter et al., 2022; Majerczyk et al., 2022). After polyI:C or LPS bind to TLRs on the surface of macrophages, they promote macrophage differentiation to a pro-inflammatory phenotype (Liu et al., 2017). Pro-inflammatory macrophages produce pro-inflammatory cytokines via nuclear factor-κB (NF-κB), which targets and upregulates the expression pro-inflammatory cytokines, including IL-1β, IL-6, and TNF-α (Liu et al., 2017). Additionally, pro-inflammatory macrophages promote the differentiation of T helper cells 17 (T_H_17), which are responsible for mediating inflammation and producing IL-17a (Liu et al., 2017) (Figure 1).

### 2.1 Interleukin-6

Macrophages, located in the maternal peripheral tissues, such as the spleen, gut, and lungs, secrete IL-6 through the NF-κB pathway in response to infection, stimulating protein synthesis, expansion of neutrophils, and B cell growth as part of a healthy immune response (Kathuria et al., 2022). However, significantly elevated levels of IL-6 have been associated with a more severe sickness response (Kathuria et al., 2022). During a healthy pregnancy, *IL-6* is upregulated during implantation and later takes part in placenta formation, early gestation, and partition (Zawadzka et al., 2021). In the fetus, IL-6 contributes to the development of the CNS by modulating neuronal and glial cell growth and survival (Parker-Athill and Tan, 2010). IL-6 can enter the placenta by directly crossing the placental barrier, but can also be endogenously induced by TNF-α, IFN-γ, IL-17a, and IL-1β (Zawadzka et al., 2021; Dahlgren et al., 2006; Zaretsky et al., 2004). The ability of IL-6 to infiltrate the placenta is modulated across pregnancy, with one MIA study on rats demonstrating higher concentrations penetrating at mid-gestation as opposed to late-gestation (Dahlgren et al., 2006). Both maternally and endogenously produced IL-6 can penetrate the blood–brain barrier (BBB) of the fetal brain and alter the cytokine balance in the brain (Zawadzka et al., 2021; Dahlgren et al., 2006; Zaretsky et al., 2004). Studies have supported the presence of elevated IL-6 in maternal serum and fetal brains acutely after MIA as an indicator of inflammatory response (Meyer et al., 2006; Hsiao and Patterson, 2011).

While many pro-inflammatory cytokines are elevated during MIA, IL-6 is believed to play a pivotal role in modulating aberrant fetal neurodevelopment, as seen through offspring behavioral evaluation. Exposure to injected IL-6 alone has been demonstrated to cause behavioral abnormalities in rodent offspring (Smith et al., 2007; Wu et al., 2017). Additionally, offspring of dams exposed to co-administration of anti-IL-6 in a polyI:C MIA model and *IL-6* knockout models exposed to polyI:C during pregnancy did not show the same behavioral deficits and displayed normalized gene expression (Smith et al., 2007; Wu et al., 2017). Additionally, while *IL-6* can be induced by the upregulation of other cytokines, this was not sufficient to induce MIA-like effects, indicating that there may be a minimum concentration threshold necessary to elicit MIA (Smith et al., 2007). One mouse study further analyzed this threshold assumption, determining that approximately 10,000 pg/mL at 2.5 h after intraperitoneal injection with polyI:C on E12.5 was a reliable threshold for inducing MIA-like behaviors (Estes et al., 2020). This concentration threshold assumption is also supported by studies comparing mid-gestation and late-gestation MIA induction. Placental penetration of IL-6 has been found to be reduced in later stages of pregnancy compared with mid-pregnancy, and in turn, behavioral deficits in late-induction MIA tend to be sub-threshold and IL-6 concentrations are lower in the fetal brain (Meyer et al., 2006; Dahlgren et al., 2006; Guma et al., 2022). The placenta may also provide a key function for IL-6 signaling, as shown by a mouse study, where the lack of IL-6 signaling in trophoblasts was sufficient to block the MIA-induced inflammatory response in the placenta and fetal brain (Wu et al., 2017).

At the level of the fetal brain, one study found that direct injection of IL-6 into the fetal ventricles of mice on E15 led to increased glutamatergic synapse density and lifelong hyperconnectivity, especially in the hippocampus (Mirabella et al., 2021). These results imitate what was seen to occur when dams’ endogenously produced IL-6 infiltrated the fetal brain (Mirabella et al., 2021). Cytokines such as IL-6 can be produced endogenously by microglia and become upregulated in pathological conditions in which microglial reactivity is heightened (Blank and Prinz, 2017). *In vitro* studies have shown that neuron-derived IL-6 promotes primary microglial proliferation (Streit et al., 2000), motility, and phagocytic activity in rats and mice (Verdillo et al., 2024), but in MIA, IL-6 can also be of maternal origin. One study looking at inflammation after stroke in mice found that, while neurons contributed to the upregulation of IL-1β, IL-6, and TNF-α, microglia were upregulating these cytokines to a much higher degree (Bourne et al., 2025). Mattei et al. (2017) found a decrease in microglial phagocytosis and an increase in IL-6 production in the hippocampus of adult male offspring mice after E15 polyI:C, while their earlier study, also in the adult male hippocampus after E15 polyI:C in rats, reported increased microglial TNF-α and IL-1β production and decreased neurogenesis (Mattei et al., 2017; Mattei et al., 2014). Another study examining fetal microglia found that IL-6 levels increased after both E12 and E15 polyI:C, whereas IL-1β levels increased only after E15 polyI:C, TNF-α levels decreased only for E12 polyI:C, and neither timepoint produced a change in IL-17 levels (Ozaki et al., 2020).

### 2.2 Interleukin 17a

IL-17a is another key cytokine in the mechanism of MIA. T_H_17 cells are a type of CD4^+^ cell located primarily at mucosal barriers, such as the gut and lungs, that predominantly produce IL-17a (Choi et al., 2016). A key factor in T_H_17 cell differentiation is IL-6, and studies have shown that IL-6 induces IL-17a expression in the placenta (Zawadzka et al., 2021; Choi et al., 2016). IL-6 stimulation activates the signal transducer and activator of transcription 3 (STAT3), which is then phosphorylated to regulate transcription of target genes, including IL-17a and retinoic acid receptor-related orphan receptor-γ (ROR-γt), which is essential for T_H_17 cell differentiation (Tripathi et al., 2017). When STAT3 is overexpressed, it leads to increased production of IL-17a (Tripathi et al., 2017). In the early stages of inflammation, IL-17a helps to increase the inflammatory response by promoting the production of other pro-inflammatory cytokines, such as IL-6, IL-1β, and TNF-α (Choi et al., 2016).

In MIA models, IL-17a has been shown to be vital in the process for the effects of MIA to occur in offspring. One polyI:C MIA model found that *IL-6* knockouts failed to increase serum IL-17a levels, supporting the need for IL-6 for IL-17a upregulation (Choi et al., 2016). This study also found that *IL-17a* mRNA was greatly upregulated in polyI:C-injected mothers compared to PBS in cultured placenta- and decidua-associated mononuclear cells isolated at E14.5 (Choi et al., 2016). Meanwhile, these cells expressed similar amounts of IL-6 (Choi et al., 2016). This study found that IL-17a induction was specific to the placenta and decidua, as they found no additional IL-17a secretion from small intestine mononuclear cells (Choi et al., 2016). This may suggest that *IL-17a* is not upregulated in the maternal periphery, but specifically in placental tissues. Regardless, if maternal *IL-17a* is upregulated then both T_H_17 cells and IL-17a produced by the mother can cross the placenta (Zawadzka et al., 2021). Once in the placenta, IL-17a can cross the early BBB, which is still forming between E11 and E17 in mice, and enter the fetal brain (Fujitani et al., 2022). Blocking *IL-17a* with anti-IL-17a injections in dams prevented the development of ASD-like symptoms, preserved cortical organization, and suppressed the MIA-induced increase in IL-17 receptor A (*IL-17RA*) mRNA expression in the fetal brain (Choi et al., 2016).

Under normal conditions, the role of *IL-17a* in fetal brain development is not well understood, but its expression levels are very low (Fujitani et al., 2022). Receptors for IL-17a are present in the cortical plate of the embryonic mouse brain, providing a pathway for their function in this region (Choi et al., 2016). Specifically, mRNA for *IL-17RA*, but not subunit C, was strongly upregulated in fetal brains at E14.5 following polyI:C-induced MIA (Choi et al., 2016). It is believed that both receptor subunit A and C must form a heterodimer for effective IL-17a signaling, but subunit A binds IL-17a first, causing conformational changes leading to the interaction between subunits (Choi et al., 2016). Downstream pathways of IL-17a lead to activation of NF-κB, resulting in the induction of other pro-inflammatory cytokines (Choi et al., 2016).

Cortical disorganization has been found in the brains of patients with ASD, thus further making this a region of interest to study mechanisms and further supporting the importance of IL-17a (Choi et al., 2016). In one study, exposure of IL-17a in the mouse fetal brain caused ASD-like behaviors, cortical disorganization, and patch-like cortical dysplasia (Fujitani et al., 2022). Injection of IL-17a into the ventricles of the fetal brain of mice at E14.5 in the absence of MIA resulted in similar outcomes in cortical disorganization, while these changes were blocked in *IL-17RA* knockout fetuses, and were not observed when IL-6 alone was injected (Choi et al., 2016). This result further supports that IL-17a, but not IL-6, acts directly on the fetal brain to produce the cortical disorganization phenotype (Choi et al., 2016). However, as previously discussed, IL-6 injection in the fetal ventricles at E15 in another mouse study was sufficient to cause connectivity changes in the hippocampus, indicating these cytokines may contribute to changes uniquely across different brain regions (Mirabella et al., 2021).

In another study, recombinant IL-17a injected into the lateral ventricle of mice at E14.5 increased the number of microglia expressing CD68, a lysosomal marker, indicating higher levels of phagocytic activity, by E18.5 in the medial region of the cerebral cortex (Sasaki et al., 2020). Microglia have been shown to express IL-17RA as one of its surface receptors, suggesting microglial dysregulation may be the downstream effector of increased IL-17a concentrations (Sasaki et al., 2020). Microglia accumulated in the medial cerebral cortex by E18.5, a region that has been shown to display patches of disorganized cortical cytoarchitecture at E18.5 in polyI:C-induced MIA fetal brains and maintained throughout adulthood (Choi et al., 2016; Sasaki et al., 2020). This may suggest that microglia are contributing to this alteration in cytoarchitecture through IL-17a signaling, which may increase microglial phagocytosis.

IL-17a has been shown to propagate neuroinflammation by inducing microglia to release excessive amounts of TNF-α, IL-6, and IL-1β in the adult brain (Zheng and Luo, 2025). In the healthy brain, microglia express mRNA transcripts for IL-1β, IL-6, and TNF-α, with exposure to LPS increasing the expression of TNF-α and IL-1β (Lee et al., 2002). One mouse study found microglia expressed IL-17a receptors and IL-6 receptors at high levels compared with other cells in the cortex, analyzed from E12.5 through postnatal day (P)1 (LaMonica Ostrem et al., 2024). Downstream signaling pathways for both IL-6 and IL-17a receptors involve NF-κB, which triggers elevated release of pro-inflammatory cytokines from microglia (LaMonica Ostrem et al., 2024).

### 2.3 IL-1β and TNF-α

IL-1β is a pro-inflammatory cytokine expressed in many cells, including microglia (Rosenzweig et al., 2014). It is elevated in both maternal serum and fetal brains after MIA induction (Simões et al., 2018; Cieślik et al., 2020). Unlike IL-6 and IL-17a, IL-1β cannot cross the placental barrier, so it is not likely that fetal increases of IL-1β are of maternal origin; however, IL-1β can cross the BBB, so it is possible it could be produced in the placenta, the fetus, or the fetal brain (Zawadzka et al., 2021). Cytokines that can cross the placenta, such as IL-6 and IL-17a, are capable of upregulating IL-1β, and, once upregulated, IL-1β can also induce its own production (Zawadzka et al., 2021). IL-1β has been found in excessive amounts in the fetal brain of MIA exposed rats, as well as in the maternal serum, indicating it has a mechanism for infiltrating and becoming upregulated in the fetal brain, where it can inhibit neural progenitor cell proliferation (Meyer et al., 2006; Zawadzka et al., 2021).

TNF-α is crucial to successful pregnancy outcomes, but the concentrations of TNF-α must be tightly regulated (Siwetz et al., 2016). The levels of TNF-α increase in the mother’s blood throughout pregnancy, but polyI:C-induced MIA studies in mice have shown that TNF-α levels are further upregulated, possibly disturbing homeostasis (Meyer et al., 2006). Similar to IL-1β, TNF-α does not cross the placenta, but has been found to be elevated in the maternal serum, though not in the fetal brain (Meyer et al., 2006; Zaretsky et al., 2004). Elevated concentrations of TNF-α and IL-1β have been demonstrated to damage the placenta, especially during early stages of pregnancy (Carpentier et al., 2011).

### 2.4 Microglia and ASD

Many studies have affirmed that MIA leads to an increased risk for neurodevelopmental disorders such as schizophrenia and ASD in offspring, but comparatively few have examined microglial outcomes, which may provide a pathophysiological mechanism. As previously discussed, microglia have receptors for important cytokines, such as IL-17a and IL-6, which lead to disorganization in the brain’s circuits. One study by Hui et al. (2018) induced MIA at E9.5 with systemic polyI:C and reported sex-specific effects on microglial morphology, ultrastructure, and distribution in the mouse dentate gyrus (DG) during adulthood. Microglia in adult male MIA-exposed offspring were more densely distributed with increased synaptic contacts and showed a tendency toward reduced phagocytic activity (Hui et al., 2018). Meanwhile, microglia in adult female MIA-exposed offspring were less dense, more phagocytic, and more often in contact with myelinated axons (Hui et al., 2018). A follow-up study by Hui et al. (2020) also found increased microglial CD68 expression in the DG of adult female offspring mice after E9.5 polyI:C, supporting a sex-specific effect of MIA on phagocytic activity. Some studies have reported that microglial density increases after MIA in various brain regions (Juckel et al., 2011; Van den Eynde et al., 2014), but others have found no significant changes in microglial density or morphology after MIA (Ozaki et al., 2020; Giovanoli et al., 2016). Likely, methodological differences between studies (e.g., gestational age of MIA induction, polyI:C dosage, brain region examined, and mouse strain) contributed to these seemingly conflicting results, given the pronounced sensitivity of microglia to environmental factors. Further, while changes to microglia density and distribution may be inconsistent, the morphological changes to microglia are clearer. Multiple studies have noted microglia in the DG of MIA offspring show microglia have reduced arborization and ramification, reflecting a more pro-inflammatory, disease-associated state (Hui et al., 2018; Carrier et al., 2024; Guneykaya et al., 2023).

It has been suggested that MIA causes microglial development to accelerate prematurely, leading to dysfunction and subsequently the onset of neurodevelopmental disorders (Matcovitch-Natan et al., 2016). A single-cell RNA sequencing study by Matcovitch-Natan et al. (2016) identified distinct stages of microglial development in mice and found that mid-gestational (E12.5 or E14.5) polyI:C-induced MIA resulted in microglia adopting a more mature transcriptional phenotype ahead of time. Given other differing effects on brain development between MIA induction timepoints, it is unsurprising that microglial transcriptional signatures are also influenced by induction timing in preclinical studies (Loewen et al., 2023). For instance, Hayes et al. (2022) reported that E9.5 polyI:C injection led to reduced expression of phagocytosis-related genes and interferon signaling in whole-brain microglia of offspring mice at P4 and in cortical and striatal microglia of adult offspring after a secondary stimulation with LPS. Conversely, a recent study also utilizing E9.5 polyI:C reported increased microglial phagocytic activity in mouse somatosensory cortices beginning after 2 weeks of age (Yan et al., 2024).

Overall, it is clear that MIA influences microglial behavior in multifaceted and context-dependent ways. In the context of treating MIA, microglia act as a key marker for potential changes throughout the brain, complementing behavioral changes. Further, when testing anti-inflammatory treatments in the mother, it is important to recognize these treatments will not penetrate through to the offspring, thus, if cytokines can still sufficiently penetrate from the mother, then microglia will upregulate cytokine production in the offspring and lead to neurodevelopmental alterations. Thus, to validate studies that aim to prevent MIA, the maternal cytokine profile and fetal neurodevelopmental outcomes should be assessed in tandem.

## 3 Vagus nerve-mediated anti-inflammatory pathways

The VN modulates the immune response through the cholinergic anti-inflammatory pathway, the spleen-mediated immune response—a unique pathway that connects the VN to the spleen—as well as the humoral immune pathway, which is mediated by the sympathetic nervous system.

Originally proposed by Tracey (2002), the cholinergic anti-inflammatory pathway picks up pro-inflammatory signals in the afferent VN fibers, which are integrated in its associated brain regions, then sends out signals along efferent VN fibers to regions of innervation, including the lungs, heart, and gut (Gargus et al., 2025; Tracey, 2002). The primary neurotransmitter released by the VN is ACh, which has interactions with the α7 nicotinic acetylcholine receptor (α7nAChR) on macrophages (Tracey, 2002). This pathway leads to the inhibition of the NF-κB pathway, reducing the release of pro-inflammatory cytokines such as TNF-α, IL-1β, and IL-6 (Gargus et al., 2025; Liu et al., 2024; Tracey, 2002).

Another part of this pathway links to the celiac superior mesenteric ganglion complex, where the VN synapses to the splenic nerve (Gargus et al., 2025; Tracey, 2002). From here, the exact mechanisms are still in debate, but the widely accepted pathway postulates that norepinephrine is released from the splenic nerve into the spleen (Pavlov and Tracey, 2012). This norepinephrine binds to adrenergic receptors on the surface of memory CD4^+^ choline acetyltransferase-expressing (ChAT) T cells to trigger the downstream synthesis of acetylcholine (ACh) (Rosas-Ballina et al., 2011). Specifically, this study noted the CD4^+^ CD44^high^ CD62L^low^ ChAT T cell population to be primarily involved (Rosas-Ballina et al., 2011). Bone marrow-derived immune cells, such as macrophages, monocytes, and dendritic cells, express α7AChR, which can be bound by ACh to begin a signaling cascade that inhibits NF-κB (Pavlov and Tracey, 2012; de Jonge et al., 2005). As previously discussed, common methods for inducing MIA trigger the NF-κB signaling pathway, resulting in the production of cytokines, including TNF-α, IL-1β, and IL-6.

Alternative hypotheses stipulate that norepinephrine is produced by the splenic nerve that binds directly to macrophages to reduce pro-inflammatory signaling (Simon et al., 2023), however, other studies have found that pro-inflammatory cytokines were not effectively reduced by stimulation of the VN in mice lacking nicotinic receptors (Pavlov et al., 2003). Other studies have suggested the VN, which uses ACh for signal transduction, directly produces it in the spleen, but there have been few supporting studies finding a direct linkage of the VN to the spleen (Gargus et al., 2025). Downregulation of inflammation may also occur at other sites of VN innervation, such as the stomach, intestines, and lungs, where ACh produced by VN efferent fibers could bind to immune cells to decrease peripheral inflammation (Gargus et al., 2025).

An alternative pathway for VN mediated anti-inflammation is the humoral immune pathway. VN circuits within the brain connect with the hypothalamic–pituitary–adrenal (HPA) axis, a primary component of the sympathetic nervous system that stimulates the release of glucocorticoids, such as cortisol and corticosterone, as well as catecholamines like norepinephrine and anti-inflammatory cytokines, including IL-10 (Pavlov et al., 2003; Murphy and Heller, 2022). Thus, afferent stimulation of the VN can lead to increased HPA axis response as a secondary mechanism for its anti-inflammatory effects (Pavlov et al., 2003).

## 4 Vagus nerve stimulation reduces pro-inflammatory cytokine production

While the specific mechanism of action is still being clarified, many studies have supported the ability of the VN to reduce pro-inflammatory cytokine production. The landmark study for the application of VNS for anti-inflammatory treatments came from Borovikova et al. (2000), where they determined VNS decreased TNF-α in a rodent endotoxemia model. In this study, human macrophage cultures conditioned by exposure to LPS were exposed to ACh, which inhibited TNF release through post-transcriptional mechanisms via nicotinic ACh receptors (Borovikova et al., 2000). Further, these ACh-treated macrophage cultures had inhibited release of other LPS-inducible cytokines, including IL-1β, IL-6, and IL-18, but did not prevent the release of the anti-inflammatory IL-10 (Borovikova et al., 2000). To investigate the stimulation of efferent VN fibers as a suppression mechanism, they used bilateral cervical vagotomy in male Lewis rats, then stimulated the distal portion of the nerve 10 min before and after LPS (Borovikova et al., 2000). They found the rats that received stimulation had a significant reduction in serum and liver TNF, while vagotomised, unstimulated rats had an increased TNF concentration peak in both locations (Borovikova et al., 2000). As mentioned, the VN is also a part of the humoral anti-inflammatory pathway. This pathway stimulates increases in corticosterone and IL-10 through afferent VN fibers (Borovikova et al., 2000). To ensure the mechanism of TNF concentration decreases was not due to humoral mechanisms, they measured corticosterone and IL-10 concentrations and did not find these to be elevated after stimulation, indicating that these effects were due to an independent pathway (Borovikova et al., 2000). This study provided much of the basis for what we understand about the cholinergic anti-inflammatory pathway today.

Since then, many studies have continued research into VNS as a potential anti-inflammatory treatment modality (Table 1). Many studies, across human and rodent models, spanning various inflammatory conditions, have supported VNS as an effective treatment to reduce symptom severity and decrease key pro-inflammatory cytokines, including TNF-α, IL-6, and IL-1β (Koopman et al., 2016; Bonaz et al., 2016; Drewes et al., 2021; Ay et al., 2016; Lerman et al., 2016; Zheng et al., 2021; Tarn et al., 2019; Brock et al., 2017; Hawkins et al., 2017). A review by Liu et al. (2024) summarized both preclinical and clinical studies utilizing VNS for its anti-inflammatory effects, and expanded on some of the many routes hypothesized as underlying mechanisms for these actions, including those previously mentioned in this review as well as interactions with the gut-brain axis and the microbiota (Liu et al., 2024).

**Table 1:** Effects of vagus nerve stimulation (VNS) on inflammation in rodent and human studies.

Reference	VNS modality	Medical condition (Model)	Clinical outcome measures	Cytokine changes
Zheng et al. (2021)	Rodent (implanted VNS) 5 Hz, 3 V, 1 mA, 500 μs pulse duration.	Preeclampsia	Reduced high blood pressure, mitigated abnormal pregnancy outcomes	↓TNF-α ↓IL-1β ↓IL-6
Hawkins et al. (2017)	Rodent (cervical non-invasive) 5 kHz sine waves in 1 ms bursts, repeated at 25 Hz.	Episodic migraine	Reduced pain, reduced sensitization compounds	↓Inflammatory Cytokines ↓CGRP
Ay et al. (2016)	Rodent (auricular and cervical non-invasive VNS) 5 kHz sine waves in 1 ms bursts, repeated at 25 Hz, 12 V.	Ischemic stroke	Reduced Infarct size, improved functional outcome	↓TNF-α
Koopman et al. (2016)	Human (implanted VNS) 10 Hz, 0.25–2.0 mA, 250 μs pulse duration.	Rheumatoid arthritis	DAS28-CRP improvement, reduced disease severity	↓TNF-α ↓IL-1β ↓IL-6
Bonaz et al. (2016)	Human (implanted VNS) 10 Hz, 1.25 mA, 500 μs pulse duration.	Crohn’s disease	CDAI improvement, Clinical remission in some	↓TNF-α ↓IL-1β ↓IL-6
Drewes et al. (2021)	Human (auricular non-invasive) 5 kHz sine waves in 1 ms bursts repeated at 25 Hz, variable voltage up to 24 V, 60 mA, 200 μs pulse duration.	Rheumatoid arthritis	DAS28 improvement, reduced disease severity	↓TNF-α ↓IL-1β ↓IL-6
Tarn et al. (2019)	Human (cervical non-invasive) 5 kHz sine waves in 1 ms bursts, repeated at 25 Hz.	Sjogren’s syndrome	Reduced fatigue, improved immune markers	↓TNF-α ↓IL-6
Lerman et al. (2016)	Human (cervical non-invasive) 5 kHz sine waves in 1 ms bursts, repeated at 25 Hz, 24 V, 60 mA.	Healthy volunteers	N/A	↓TNF-α ↓IL-1β ↓IL-8 ↓MCP-1
Brock et al. (2017)	Human (cervical non-invasive VNS) 5 kHz sine waves in 1 ms bursts, repeated at 25 Hz.	Healthy volunteers	Elevated HRV	↓IL-6 ↓IL-17 ↓IL-23
Okdahl et al. (2024)	Human (cervical non-invasive VNS) 5 kHz sine waves in 1 ms bursts, repeated at 25 Hz, 24 V, 60 mA.	Diabetes	N/A	-IL-6 -IL-8 -IL-10 -TNF-α -IFN-γ
Kox et al. (2015)	Human (transcutaneous cervical VNS) 5 kHz sine waves in 1 ms bursts, repeated at 20 Hz, 2–10 V.	Healthy volunteers with experimental endotoxemia	No differences in body temperature, heart rate, or mean arterial pressure.	-TNF-α -IL-6 -IL-8 -IL-10
Chaudhry et al. (2019)	Human (transcutaneous cervical VNS) 5 kHz sine waves in 1 ms bursts, repeated at 20 Hz, 0–24 V.	Refractory episodic migraine, chronic migraine	Reduction in severe migraine attacks	-TNF-α -IL-6 -IL-10 -IL-1β

CDAI, Crohn’s disease activity index; CGRP, calcitonin gene-related peptide; DAS28, disease activity composite score 28-joint C reactive protein; HRV, heart rate variability; IL, interleukin; TNF-α, tumor necrosis factor alpha.

However, one meta-analysis by Schiweck et al. (2024) challenges the efficacy of VNS for anti-inflammatory treatments, finding no significant short- or long-term changes to cytokines with VNS treatment (Schiweck et al., 2024). One such study from that meta-analysis was by Kox et al. (2015), who noted no cytokine alterations in a human study of endotoxemia (Kox et al., 2015). An additional study looking at chronic migraine found transcutaneous cervical VNS could effectively decrease severe migraine attacks, but had no effect on pro-inflammatory cytokine concentrations (Chaudhry et al., 2019). Another recent study by Okdahl et al. (2024) corroborates this lack of effectiveness in isolated tests, finding transcutaneous VNS did not alter cytokine levels in a human diabetes trial (Okdahl et al., 2024). This disparity further emphasizes the need to build consistency between models and human studies, as a means to clarify the effectiveness of VNS for anti-inflammatory treatments. Currently, standards for stimulation vary between devices, conditions, and route of administration, leading to difficulty replicating results (Liu et al., 2024; Schiweck et al., 2024). By expanding our understanding of the hypothesized mechanisms of the anti-inflammatory action of VNS, we can better understand these differences in outcomes.

As previously mentioned, VNS is currently approved primarily as a treatment for refractory epilepsy and major depression disorder (Gargus et al., 2025). In these conditions, VNS is found to be effective in approximately 50% of the tested population, often classifying those patients as “responders” versus “non-responders” (Gargus et al., 2025). The process behind what makes a patient responsive or unresponsive to the therapy is not yet understood, and this may evidently help explain the apparent disparities in anti-inflammatory studies and should be investigated once models achieve a greater consistency (Gargus et al., 2025). The genetic differences underlying humans and in mouse strains are an important consideration for the kinds of cytokines induced and their associated response (Babri et al., 2014). Another avenue worth exploring in this context is the variability of the major histocompatibility complex, which accounts for the highest component for genetic risk of neurological diseases (Misra et al., 2018).

### 4.1 Safety considerations for vagus nerve stimulation during pregnancy

While not many studies have been conducted using VNS in pregnancy, some pre-clinical and clinical studies have been conducted on individuals using VNS for the treatment of epilepsy during their pregnancy. The VN is connected to the uterus as part of a system to regulate contractions and blood flow in mice (Ding et al., 2021). Most concerns come from the less direct connections the VN has to the body, however these concerns are theories based on existing pathways than proven reasons for concern and would require significantly more research to be discussed thoroughly (Ding et al., 2021).

One study used a rat pregnancy model, where surgical implantation of the VNS device was performed on E16, and dams received stimulation until birth, and the brains of the pups were collected at P0 (Judkins et al., 2018). The VNS devices delivered 5 Hz or 1,000 Hz at 1 mA amplitude with a 500 μs pulse width, occurring for 30 min, followed by 5.5 h off time, repeated four times per day (total 5–6 days of exposure) (Judkins et al., 2018). They determined that VNS did not increase pro-inflammatory cytokine levels in the medulla of pups, nor did it interfere with parturition (Judkins et al., 2018). Overall, they determined VNS did not affect the viability of offspring or impair physiological processes essential for normal birth (Judkins et al., 2018). A follow-up study used a rat model of preeclampsia, a hypertensive disorder that can occur during pregnancy, from E14.5 to E20.5, during which days they also received invasive VNS treatment following the same parameters, except only applying 5 Hz, at a voltage of 3 V (Zheng et al., 2021; Judkins et al., 2018). They found that the VNS treatment decreased systolic blood pressure in the preeclampsia model rats and mitigated abnormal pregnancy outcomes associated with preeclampsia, while also decreasing peripheral cytokines levels (Zheng et al., 2021).

In clinical cases, one small sample study (four women covering five pregnancies) that applied VNS for epilepsy had no pregnancy or birth complications that could be attributed to the treatment (Rodríguez-Osorio et al., 2017). Parameters included a 1.25–1.5 mA amplitude, 21–30 s on, 1.8–5 min off cycles (Rodríguez-Osorio et al., 2017). Another study used a retrospective database and found nine patients across 13 pregnancies, who used VNS for epilepsy during pregnancy (Vu et al., 2024). VNS amplitude ranged from 0.5 mA to 1.5 mA (Vu et al., 2024). They found no major congenital malformations, and noted complications observed aligned with those previously described during pregnancy for patients with epilepsy, however they acknowledge the data to be limited (Vu et al., 2024). Another study analyzed a database consisting of four women and seven pregnancies and noted obstetric complications in three patients, which required c-section (Suller Marti et al., 2019). They further noted one child had intellectual disabilities of unclear severity, while all other babies were healthy (Suller Marti et al., 2019). In these cases, VNS parameters ranged from 0.75 mA to 3.5 mA (Suller Marti et al., 2019). In another case where VNS was applied for the treatment of depression, a case study found the treatment was safe for the mother and fetus (Husain et al., 2005). This patient received VNS at an amplitude of 0.25 mA, with a 20 Hz signal frequency, and a 250 μs pulse width with a 30s on, 5 min off cycle (Husain et al., 2005). A mini review by Ding et al. (2021) summarizes these known cases of VNS during pregnancy (Ding et al., 2021). They note two cases of miscarriages across 44 pregnancies, which were suggested as possibly being attributed to antiepileptic drugs, but cannot be made certain (Ding et al., 2021). A further 2 cases among successful pregnancies had pre-eclampsia (Ding et al., 2021). No other maternal concerns were noted (Ding et al., 2021). In terms of fetal outcomes, they reported one of the 42 surviving fetuses had severe fetal malformations, but the mother had also been taking four antiepileptic drugs, while another case of fetal malformations were found in a mother who was taking three antiepileptic drugs (Ding et al., 2021). Because of the presence of these drugs, it is difficult to discern the cause of these malformations (Ding et al., 2021).

Generally, VNS is an effective treatment for maternal conditions such as epilepsy and major depression and studies report more favorable evidence than adverse for the pregnancy and fetus. However, these studies represent very limited populations, and there are no long-term clinical studies on the outcomes of VNS in offspring development (Rodríguez-Osorio et al., 2017; Vu et al., 2024; Husain et al., 2005). Further, these cases can be confounding, as epilepsy and depression can also affect neurodevelopmental outcomes in the offspring and make it difficult to target the cause (Rodríguez-Osorio et al., 2017; Vu et al., 2024; Husain et al., 2005). Further research on this topic should be explored in pre-clinical models to assess the potential impacts in a more controlled environment, and to follow up on long-term outcomes for the offspring, which is lacking from all aforementioned studies.

## 5 Conclusion

The prenatal period of fetal development is a vulnerable time for the development of neurodevelopmental disorders such as ASD, where alterations can be prevented rather than corrected. The mechanisms of MIA have been closely connected to maternal cytokine production, importantly implicating *IL-6* as an upstream target and *IL-17a* as a downstream mediator of fetal brain modulation. Additionally, fetal brain microglia may contribute to MIA through their response to cytokine alterations, contributing to changes in neurons, synaptic structures and exacerbating production of pro-inflammatory cytokines. While prenatal treatment with *IL-6* and *IL-17a* blockades proves effective for behavioral symptom mitigation in rodent models, they are not likely candidates for translational application. Meanwhile, VNS has suggested to be safe for the maternal and gestational outcomes and has also been demonstrated to be a potent anti-inflammatory therapy. Further elucidation of the mechanisms of VNS for anti-inflammatory actions and MIA-induced neurodevelopmental changes in offspring will be crucial in pinpointing this therapy as a potential preventative strategy. Studies should focus on the maternal and prenatal mechanisms to better understand the route of action, while using changes to offspring behavioral and long-term neurodevelopment as a verification of effective prevention. Research should also critically analyze the long-term effects of VNS on the offspring of exposed mothers to ensure neurodevelopmental processes are not altered by the treatment itself.
